# Isolation and Genome Characterization of the Virulent *Staphylococcus aureus* Bacteriophage SA97

**DOI:** 10.3390/v7102870

**Published:** 2015-10-01

**Authors:** Yoonjee Chang, Hakdong Shin, Ju-Hoon Lee, Chul Jong Park, Soon-Young Paik, Sangryeol Ryu

**Affiliations:** 1 Department of Food and Animal Biotechnology, Department of Agricultural Biotechnology, Research Institute of Agriculture and Life Sciences, and Center for Food and Bioconvergence, Seoul National University, Seoul 151-921, Korea; yjchang10@snu.ac.kr (Y.C.); hakdong.shin@gmail.com (H.S.); 2 Department of Food Science and Biotechnology, Kyung Hee University, Yongin 446-701, Korea; juhlee@khu.ac.kr; 3 Department of Dermatology, College of Medicine, the Catholic University of Korea, Seoul 137-701, Korea; cjpark777smp@gmail.com; 4 Department of Microbiology, College of Medicine, the Catholic University of Korea, Seoul 137-701, Korea; paik@catholic.ac.kr

**Keywords:** bacteriophage, genome analysis, *Staphylococcus aureus*

## Abstract

A novel bacteriophage that infects *S. aureus*, SA97, was isolated and characterized. The phage SA97 belongs to the *Siphoviridae* family, and the cell wall teichoic acid (WTA) was found to be a host receptor of the phage SA97. Genome analysis revealed that SA97 contains 40,592 bp of DNA encoding 54 predicted open reading frames (ORFs), and none of these genes were related to virulence or drug resistance. Although a few genes associated with lysogen formation were detected in the phage SA97 genome, the phage SA97 produced neither lysogen nor transductant in *S. aureus*. These results suggest that the phage SA97 may be a promising candidate for controlling *S. aureus*.

## 1. Introduction

Bacteriophages are natural antibacterial agents that lyse a specific bacterial host strain [1]. Based on their bacterial host specificity and bacteriolytic activity, the use of bacteriophages has been suggested as a potential biocontrol strategy for various pathogenic bacteria. *Staphylococcus aureus* is a major foodborne and clinical pathogen that causes food poisoning, endocarditis, staphylococcal scalded skin syndrome and toxic shock syndrome [2]. Furthermore, the emergence of antibiotic-resistant *S. aureus*, including methicillin-resistant *S. aureus* (MRSA) [3], has complicated the treatment of *S. aureus* infections [4]. Bacteriophage treatment could be an alternative method for controlling *S. aureus*. Various bacteriophages have been used as alternative antibacterial therapies for human patients in eastern Europe for several decades [5]. In addition, the US Food and Drug Association (FDA) has approved the application of bacteriophages as a safe food preservative [6]. 

Virulent phages are generally preferred as biocontrol agents as they cannot integrate their genome into the bacterial chromosome to form lysogens and will always lyse and kill infected target bacterial cells [7]. Staphylococcal lytic phages have demonstrated their potential as biocontrol agents of food [1] and phage therapy of human or animal infections [8,9,10]. For example, Myovirus phages S25-3 [11], K [9] and the Podoviruses S13’ [11], S24-1 [11] are virulent phages that have been applied to food samples or mice. However, most reported *S. aureus* phages are siphoviruses and known as temperate phages that contain lysogeny modules in their genomes [12,13]. Enterobacteria phage lambda [14,15,16] and *S. aureus* phage phi11 [14] are well-known temperate phages that form lysogens. These phages all contain typical lysogen modules including *cro*-like repressor, antirepressor, integrase, excisionase, *cI*-like repressor, and lambda contains additional *cII* and *cIII* regulatory proteins. Integrase mediates site-specific recombination between two DNA recognition sequences in phage and host bacteria, while excisionase is involved in excisional recombination by excising the phage genome from the bacterial chromosome. CI-like repressor represses lytic functions; Cro-like repressor, which is a repressor of *cI*, leads the phage to the lytic infection cycle, while the antirepressor blocks phage repressors. Among the lysogen formation-related genes, *cI*-like repressor gene is the key regulator that maintains the phage in the lysogenic life cycle [14,15,16].

Here, the phage SA97, which specifically infects *S. aureus* without lysogen formation, was isolated and characterized. An analysis of the whole genome of the phage SA97 revealed part of the genes encoding a lysogeny module but no genes related to virulence or drug resistance. An analysis of lysogen formation or transduction by SA97 showed that SA97 produced neither lysogen nor transductant, suggesting that the phage SA97 may be applicable as a biocontrol agent against *S. aureus.*

## 2. Materials and Methods

### 2.1. Bacterial Strains, Media, and Growth Conditions

The bacterial strains used in this study are summarized in Table 1. Food isolates 1 to 4 were from vegetable, pork, beef and Korean meal kimbab (rice with laver, ham, egg, cucumber, pickled radish and crab meat), respectively. They were isolated and purified by Baird-Parker agar medium (Difco, Detroit, MI, USA) and identified by VITEK^®^ 2 Compact (bioMerieux, Inc., Hazelwood, MO, USA). These strains were all grown in tryptic soy broth (TSB) medium (Difco) at 37 °C with agitation. Tryptic soy agar (Difco) containing 1.5% (*w/v*) agar was used to count the bacteria.

### 2.2. Bacteriophage Isolation and Propagation

Bacteriophage SA97 was isolated from the skin using the strain *S. aureus* RN4220 as a bacterial host strain. To isolate the phage, 0.5 cm^2^ of skin was rubbed with a cotton swab that was subsequently homogenized with 1 mL of sodium chloride and magnesium sulfate (SM) buffer (100 mM NaCl, 10 mM MgSO_4_, and 50 mM Tris-HCl, pH 7.5). The sample was then mixed with TSB broth supplemented with 10 mM CaCl_2_, sub-cultured with *S. aureus* RN4220 and incubated at 37 °C for 12 h with shaking. After incubation, the sample was centrifuged at 8000× *g* for 10 min and filtered to remove bacterial cells and obtain the supernatant that contained the bacteriophage. For phage propagation, TSB broth containing 10 mM CaCl_2_ was first sub-inoculated with *S. aureus* RN4220, and the culture was incubated at 37 °C for 1.5 h. Subsequently, the phage was added at a multiplicity of infection (MOI) of 1, and the culture was incubated for 3 h at the same temperature with shaking. To prepare a high-titer phage, the phages were precipitated with polyethylene glycol (PEG) 6000 and concentrated using CsCl density gradient ultracentrifugation [17]. Finally, to confirm the phage plaque formation, the supernatant was overlaid on 0.4% TSA soft agar containing 10 mM CaCl_2_ and *S. aureus* RN4220. Plaques were evident after incubation in at 37 °C for 12 h.

### 2.3. Transmission Electron Microscopy (TEM)

The phage SA97 was analyzed using transmission electron microscopy (TEM). The phage suspension was placed on a carbon-coated copper grid and negatively stained with 2% uranyl-acetate (pH 4.0). The sample was examined under an energy-filtering transmission electron microscope at an operating voltage of 120 kV [18]. Phage SA97 was identified and classified according to the guidelines of the International Committee on Taxonomy of Viruses [19]. 

### 2.4. Bacteriophage Host Range

The bacterial strains listed in Table 1 were incubated overnight at 37 °C. Each bacterial culture was added to 5 mL of the 0.4% TSA soft agar and overlaid on TSA agar plates. Subsequently, the phage SA97 containing diluted lysates was spotted onto the prepared plate and incubated at 37 °C for at least 6 h to obtain single plaques. After incubation, we could determine the infectivity based on the clarity of the spots, and efficiency of plating (EOP) was calculated by dividing the phage titer on the test strain by the phage titer on the reference strain. All the experiments were performed in triplicate.

### 2.5. Bacterial Challenge Assay

Fifty milliliters of TSB broth containing 10 mM CaCl_2_ was sub-inoculated with *S. aureus* RN4220, and the culture was incubated at 37 °C until it reached the early exponential growth phase (3.5 × 10^8^ ± 0.2 × 10^8^). The culture was then infected with the phage at a MOI of 1 or 10. The OD_600_ was measured each hour after phage infection for 15 h [20]. An un-infected culture was used as a control. All the experiments were performed in triplicate.

### 2.6. Lysogen Formation

To evaluate the ability of the phage SA97 to form lysogens, the phage-resistant bacteria were isolated 10 h after phage infection, and the presence of SA97 gene fragments in the fifty of SA97-resistant colonies was analyzed by PCR using primers specific to the transcriptional regulator gene (SA97_044) or antirepressor gene (SA97_045). In addition, the phage-resistant strains grown to exponential phase were induced with mitomycin C at a final concentration of 0.5 μg/mL and centrifuged for 5 min at 10,000 × *g* after incubation at 37 °C. The supernatants were filtered using 0.22-μm filter and spotted onto *S. aureus* overlay. 

### 2.7. One-Step Growth Curve Assay

One-step growth curve analysis was performed as previously described [21]. Briefly, phage was mixed with *S. aureus* RN4220 in its early exponential growth phase at MOI of 0.001 in the presence of 10 mM CaCl_2_ and allowed to adsorb for 10 min at room temperature, then it was centrifuged at 6000 × *g* for 10 min. The pellet containing infected cells was suspended in 50 mL of fresh TSB liquid medium and incubated at 37 °C with shaking. Two sets of samples were taken every 5 min up to 1 h. To release the intracellular phages, CHCl_3_ was added to one of them, and the eclipse period was determined. Subsequently, each sample was immediately titered by the double-layer agar plate method and latent period, eclipse period and burst size were calculated. All the experiments were performed in triplicate.

### 2.8. Receptor Analysis 

*S. aureus* RN4220, a strain free of prophages, restriction mechanisms and capsules [22,23], was used in this study (Table 1). To identify the phage receptor, we obtained the Δ*tagO*/RN4220 mutant, which is deficient in the peptidoglycan-anchored wall teichoic acid (WTA) [24], and its complemented strain using plasmid pRB474-*tagO*, which was constructed by sub-cloning the *tagO* gene into an *Escherichia coli*-*S. aureus* shuttle expression vector [25]. We carried out a spotting assay with the wild type RN4220, Δ*tagO*/RN4220 mutant and the *tagO* complemented strains. 

### 2.9. Adsorption Assay

The phage adsorption ability was assayed as previously described [26]. Overnight cultures of the bacterial strains were diluted 1:100 in TSB and incubated until the OD_600_ reached 2.0. One milliliter of the strain cultures was diluted 10-fold in fresh TSB, and the phage was added to the each diluted culture at an MOI of 0.01. The cultures were incubated at 37 °C for 10 min. A sample was collected immediately after reaction and 10 min after incubation for each culture and immediately centrifuged at 14,000 × *g* and 4 °C for 1 min. The samples were then filtered using 0.22-μm filters. Subsequently, the supernatants were serially diluted and overlaid on TSA plates to determine the titers of unabsorbed phages. All the experiments were performed in triplicate.

### 2.10. Determination of the Frequency of the Bacteriophage-Insensitive Mutants (BIMs)

The frequency of the BIMs was determined as previously described [27]. Phage was mixed with *S. aureus* (10^9^ CFU/mL) cell suspension containing 10 mM of CaCl_2_ with a MOI of 100. After incubation at 37 °C for 10 min, serial dilutions of the mixtures were plated. The BIMs frequency was determined by dividing remaining viable counts by the initial viable counts. All the experiments were performed in triplicate.

### 2.11. Transduction Assay

A transduction assay was performed to determine the ability of phage SA97 to induce horizontal gene transfer between the host bacteria cells and bacteriophages. Donor phage lysate was prepared by infecting SA97 phage to the host bacteria containing the erythromycin antibiotic-resistant cassette (Δ*ypfP*::erm, the glycolipid-deficient mutant) [28]. After infection, the cultures were incubated in TSB for 8 h at 37 °C. After incubation, the cultures were treated with chloroform, and the supernatant lysate was harvested. To determine the ability of the phage to transduce host bacteria, supernatant phage lysate was added to the recipient host bacteria culture (*S. aureus* RN4220, 10^10^ CFU/mL) at an MOI of 10 and the culture was incubated for 15 min at 37 °C. TSB containing ethylene glycol tetraacetic acid (EGTA) was added to the culture (final concentration of 20 mM) to stop the reaction. After incubation for 20 min under the same conditions, the phage-infected bacterial cells were harvested, spread on TSA agar media containing erythromycin and incubated at 37 °C. All the experiments were performed in triplicate, and the transduction frequencies were calculated as the quotient of the number of transductants divided by the number of input PFU per plate [29].

### 2.12. Bacteriophage Genomic DNA Purification

Bacteriophage genomic DNA was purified as previously described [30]. Prior to purification, the phage lysates were treated with DNase and RNaseA at 37 °C for 1 h to remove bacterial DNA and RNA contamination, respectively. The phage lysate was then treated with lysis buffer containing 0.5 M of EDTA, 10 mg/mL of proteinase K and 1% of sodium dodecyl sulfate (SDS) for 15 min at 65 °C. Finally, ethanol precipitation was performed, followed by phenol-chloroform DNA purification. 

### 2.13. Full-Genome Sequencing of Bacteriophage SA97 and Bioinformatics Analysis

The extracted SA97 phage genomic DNA was sequenced with a Genome Sequencer FLX titanium sequencer (Roche, Mannheim, Germany) and assembled with the GS de novo assembler software (Roche) at Macrogen Inc., South Korea. Open reading frames (ORFs) were predicted using the FGENESB (http://www.softberry.com), Glimmer v3.02 [31] and GeneMark.hmm [32] software packages. The ORFs were annotated using the InterProScan [33] and BLASTP [34] programs. Comparative genomic analysis of SA97 with other phietalikevirus Subgroup2 phages and visualization were conducted with BLASTN [35] and ACT12 [36]. The complete genome sequence of *S. aureus* phage SA97 was deposited in GenBank under accession number KJ716334.

## 3. Results and Discussion

### 3.1. Phage SA97 Isolation and Its Morphological Analysis

**Figure 1:**
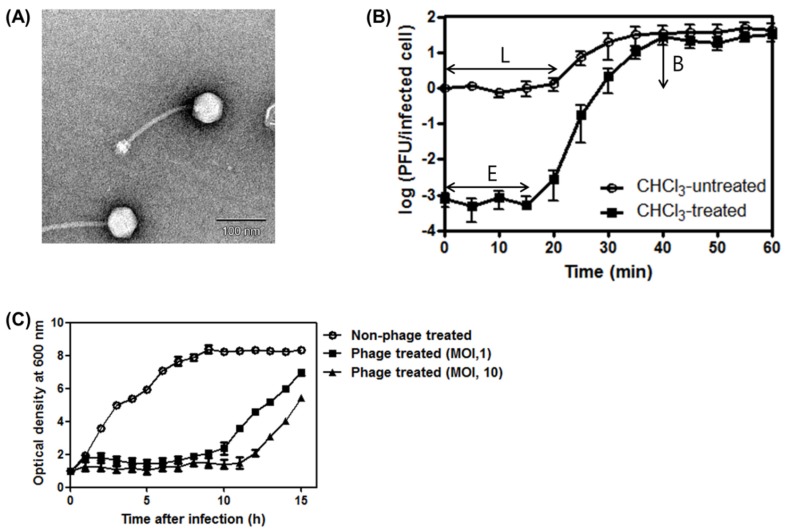
General characterizations of bacteriophage SA97. (**A**) Electron micrograph of phage SA97. The phage belongs to the *Siphoviridae* family. The scale bar represents 100 nm; (**B**) One-step growth curve analysis of phage SA97 on exponential culture of *S. aureus* RN4220 incubated in tryptic soy broth (TSB) medium at 37 °C under agitation. Cells were chloroform treated (○) or untreated (■). E, eclipse period; L, latent period; B, burst size; (**C**) Inhibition assays of *S. aureus* RN4220 with phage SA97 at 37 °C. ○, control group without phage; ■, ▲, experimental groups with phage SA97 (MOI of 1 and 10, respectively). The data shown are the mean values from three independent measurements and the error bars represent the standard deviations.

The *S. aureus* infecting bacteriophage SA97 was newly isolated. This phage formed clear plaques against the *S. aureus* RN4220 bacterial host strain. Transmission electron microscopy (TEM) revealed an icosahedral capsid (63 ± 2 nm, *n* = 5) with a long, flexible and non-contractile tail (171 ± 6 nm, *n* = 5) for phage SA97, indicating that this phage belongs to the *Siphoviridae* family (Figure 1A). The baseplate structure of the phage SA97 consists of multiple disc baseplates and is similar to those of phages StB12, StB27 and StB20 [37]. However, the tail of phage SA97 is shorter than those of the above-mentioned phages (206 nm for StB12, 213 nm for StB27, and 250 nm for StB20) [37]. According to Xia and Wolz [38], phage SA97 belongs to serogroup B, which contains phages with a tail length of less than 200 nm.

### 3.2. One-Step Growth Curve of SA97and Bacterial Challenge Assay

The one-step growth kinetics of SA97 showed a latent period time of 20 min, an eclipse period time of 15 min and a burst size of 32 PFU/infected cell when infecting *S. aureus* RN4220 (Figure 1B).

To determine the bacteriolytic activity of the phage SA97, a bacterial growth inhibition assay was performed in the presence of SA97 (Figure 1C). The growth inhibition of *S. aureus* by SA97 persisted for up to 10 or 11 h after infection with MOI of 1 or 10, respectively. SA97 showed a longer inhibitory effect than most other *S. aureus* phages such as CS1, DW2 [10] and SAH-1 [39], all of which showed only 3 h of growth inhibition. These results demonstrate that SA97 possesses strong bacteriolytic activity, which is essential in phage therapy. Re-growth of the culture 10 h after infection with SA97 suggested the possibility of development of lysogen or resistant mutant. However, PCR analysis of the 50 *S. aureus* colonies resistant to SA97 obtained 10 h after phage infection using primers specific to the transcriptional regulator gene (SA97_044) or antirepressor gene (SA97_045) detected neither gene. In addition, no plaques were observed after induction of the resistant strains with mitomycin C, indicating that the resistant strains were not SA97 lysogens but resistant mutants.

### 3.3. Receptor Analysis

**Figure 2:**
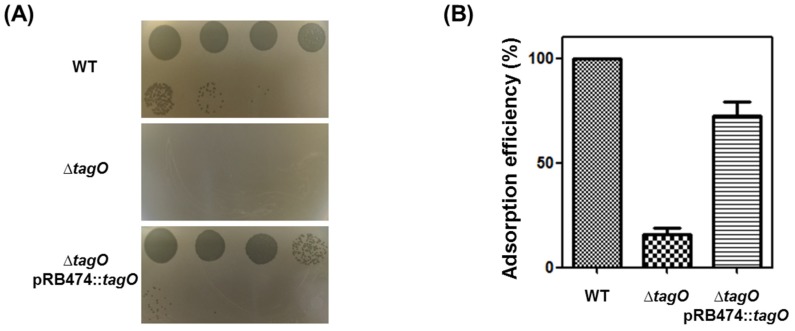
(**A**) Wall teichoic acid (WTA)-dependent infection of *S. aureus* phage SA97. The phage SA97 lysate was spotted onto lawns of wild-type RN4220 (WT), *ΔtagO* mutant (*ΔtagO*), and *tagO*-complemented (*ΔtagO* pBR474::*tagO*) strains. Plaque formation indicates successful adsorption and infection by the phage SA97. EOP relative to WT strain was 7.5 × 10^−2^ ± 0.2 × 10^−2^ on *ΔtagO* pBR474::*tagO* strain; (**B**) WTA-dependent adsorption ability of *S. aureus* phage SA97. The adsorption efficiency relative to the adsorption to wild-type strain RN4220, which was set to 100%, is indicated. The data shown are the mean values from three independent measurements and the error bars represent the standard deviations.

The only known host receptor of *S. aureus* phages is peptidoglycan-anchored wall teichoic acid (WTA). Xia *et al.* [40] demonstrated that WTA is required for siphovirus and myovirus infection of *S. aureus*; thus, a *ΔtagO* mutant of *S. aureus* (RN4220*ΔtagO*) [41] was tested (Figure 2A). The RN4220*ΔtagO* strain was resistant to SA97, and the phage sensitivity was recovered by complementing the strain with *tagO* [42]. The phage adsorption assay also showed that phage adsorption was severely reduced in the RN4220*ΔtagO* strain (Figure 2B), indicating that the host receptor for phage SA97 is peptidoglycan-anchored WTA. 

### 3.4. Host Range of Bacteriophage SA97

Plaque formation efficiency of the phage SA97 was determined for 19 *S. aureus* strains and several other strains of both Gram-positive and Gram-negative bacteria as shown on Table 1. SA97 phage could infect only *S. aureus* strains. Six out of 13 *S. aureus* type strains and all six *S. aureus* local isolates tested were sensitive to SA97. It is interesting to note that nine of these 12 sensitive strains were isolated in Korea, indicating that SA97 phage may be more effective for controlling *S. aureus* strains isolated in Korea. 

### 3.5. Bacteriophage SA97 Genomic Analysis

**Figure 3:**
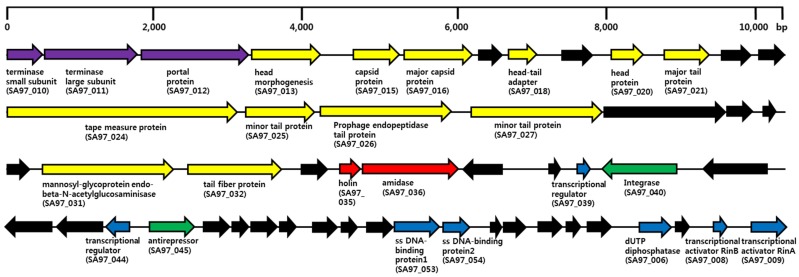
Genome map of phage SA97. Representation of the ORF (ORF 1 to 54) organization of phage SA97. The predicted genes are indicated as arrows. Blue arrows, DNA regulation module; purple arrows, packaging module; yellow arrows, phage structural proteins; red arrows, host lysis proteins; green arrows, lysis/lysogeny module; black arrows, hypothetical proteins.

The phage SA97 genome was sequenced and analyzed. The complete genome sequence of the phage SA97 revealed that the genome consists of 40,592 bp with a GC content of 34.25% encoding 54 predicted open reading frames (ORFs) and no tRNA genes (Figure 3).

The functional analysis of the predicted ORFs revealed that they can be classified into five functional groups: phage structure (major capsid protein, head morphogenesis protein, head-tail connector protein, major and minor tail proteins, tape measure protein, minor structure proteins, tail fiber protein and endo-β-*N-*acetylglucosaminidase), packaging (portal protein and terminase small/large subunits), host lysis (holin and endolysin), DNA regulation (single-stranded DNA-binding protein, dUTP diphosphatase, transcriptional activators RinAB and transcriptional regulators) and lysis/lysogeny module (integrase and antirepressor) (Table 2). 

**Table 1:** The antimicrobial spectrum of bacteriophage SA97

Bacteria	Plaque Formation of SA97^a^	E.O.P ^b^	Reference or Source ^c^
***Staphylococcus strains***			
*Staphylococcus aureus* RN4220	C	1.0 × 10^0^ ± 0.0 × 10^0^	[42]
*Staphylococcus aureus* KCTC 1916	C	1.1 × 10^0^ ± 1.8 × 10^−1^	KCTC
*Staphylococcus aureus* ATCC 29213	C	1.7 × 10^−4^ ± 2.1 × 10^−5^	ATCC
*Staphylococcus aureus* ATCC 33593	C	1.2 × 10^0^ ± 8.7 × 10^−2^	ATCC
Methicillin resistant *Staphylococcus aureus* CCARM 3089	C	1.0 × 10^0^ ± 8.1 × 10^−2^	CCARM
Methicillin resistant *Staphylococcus aureus* CCARM 3090	C	7.4 × 10^−2^ ± 3.1 × 10^−2^	CCARM
*Staphylococcus aureus* food isolate 1 (from vegetable)	C	4.9 × 10^−2^ ± 1.6 × 10^−2^	This study
*Staphylococcus aureus* food isolate 2 (from pork)	C	1.2 × 10^0^ ± 1.0 × 10^−1^	This study
*Staphylococcus aureus* food isolate 3 (from beef)	C	1.2 × 10^0^ ± 2.8 × 10^−1^	This study
*Staphylococcus aureus* food isolate 4 (from Kimbab)	C	1.0 × 10^0^ ± 4.0 × 10^−1^	This study
*Staphylococcus aureus* clinical isolate 1	C	2.0 × 10^−4^ ± 5.0 × 10^−5^	This study
*Staphylococcus aureus* clinical isolate 0055	C	1.1 × 10^−3^ ± 5.5 × 10^−5^	GNUH-NCCP
*Staphylococcus aureus* Newman	-	-	[43]
*Staphylococcus aureus* ATCC 6538	-	-	ATCC
*Staphylococcus aureus* ATCC 23235	-	-	ATCC
*Staphylococcus aureus* ATCC 25923	-	-	ATCC
*Staphylococcus aureus* ATCC 12600	-	-	ATCC
*Staphylococcus aureus* ATCC 13301	-	-	ATCC
*Staphylococcus aureus* ATCC 27664	-	-	ATCC
*Staphylococcus xylosis* ATCC 29971	-	-	ATCC
*Staphylococcus cohnii* ATCC 29974	-	-	ATCC
*Staphylococcus warneri* ATCC 10209	-	-	ATCC
*Staphylococcus intermedius* ATCC 29663	-	-	ATCC
**Other Gram positive bacteria**			
*Enterococcus faecalis* ATCC 29212	-	-	ATCC
*Bacillus cereus* ATCC 14579	-	-	ATCC
*Bacillus subtilis* ATCC 23857	-	-	ATCC
*Listeria monocytogenes* ATCC 19114	-	-	ATCC
**Gram negative bacteria**			
*Salmonella enterica serovar* Typhimurium SL1344	-	-	ATCC
*Escherichia coli* MG1655 ATCC 47076	-	-	ATCC
*Escherichia coli* O157:H7 ATCC 35150	-	-	ATCC
*Cronobacter sakazakii* ATCC 29544	-	-	ATCC
*Pseudomonas aeruginosa* ATCC 27853	-	-	ATCC

^a^ C, clear plaques; -, no plaque. ^b^ The EOP is relative to an average result obtained for RN4220. ^c^ ATCC, American Type Culture Collection; KCTC, Korean Collection for Type Cultures; GNUH-NCCP, Gyeongsang National University Hospital Branch of National Culture Collection for Pathogens; CCARM, Culture Collection of Antimicrobial Resistant Microbes.

Interestingly, three genes in the category of host lysis may be responsible for the host lysis activity of phage SA97. Holin (SA97_035) is generally known to create a hole in the host inner membrane to expose the host peptidoglycan layer to endolysin. Endolysin (SA97_036) may then lyse the host peptidoglycan to allow phage burst-out. In general, Gram positive endolysins form in a modular structure, consisting of enzymatically active domains (EADs) and a cell wall binding domain (CBD) [44] which leads the enzyme specifically to the substrate bacterial peptidoglycan. The BLASTP analysis of endolysin revealed two major protein domains containing CHAP (PF05257) and amidase_3 (PF01520), which may be involved in host lysis via the activities of endopeptidase and amidase. However, a host cell wall-binding domain (e.g., SH3 domain) was not detected, suggesting that this phage endolysin may contain a new type of CBD. Endo-β-*N-*acetylglucosaminidase (SA97_032), which is known as a tail-associated cell wall hydrolase, may degrade the peptidoglycan of the bacterial cell wall during infection [45]. 

A few putative genes associated with lysogen formation [antirepressor (SA97_045) and integrase (SA97_040)] were detected in the category of DNA manipulation and regulation; however, the major components of the lytic/lysogenic decision gene cluster, *cI*- or *cro*-like repressors, were missing. 

**Figure 4:**
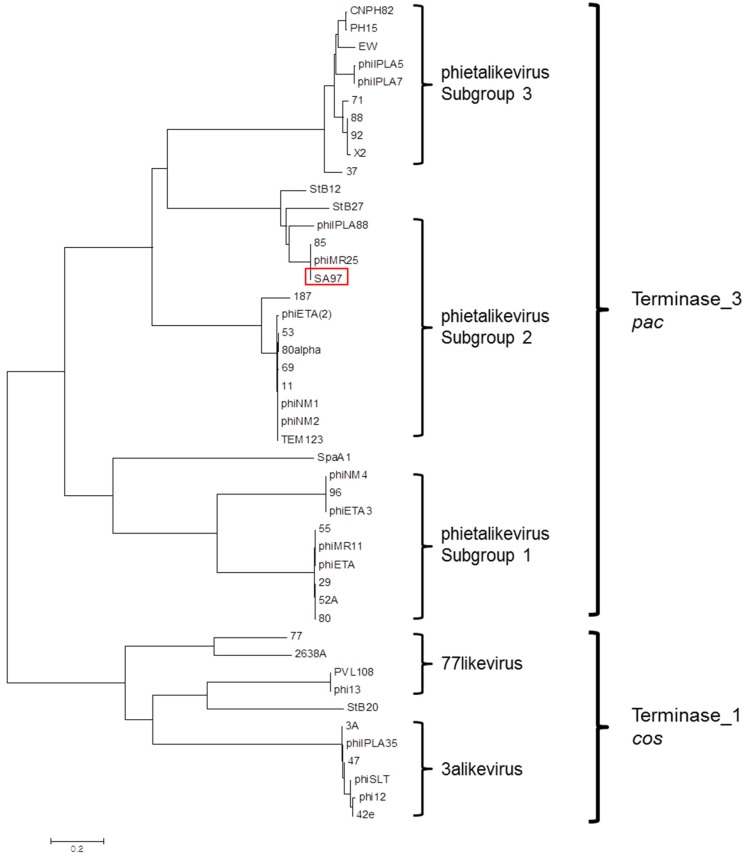
Phylogenetic analysis of terminase large subunits in the staphylococcal bacteriophages. The terminase large subunits were compared using the ClustalW program, and the phylogenetic tree was generated with the neighbor-joining method with *P* distance values using the MEGA5 program.

**Figure 5:**
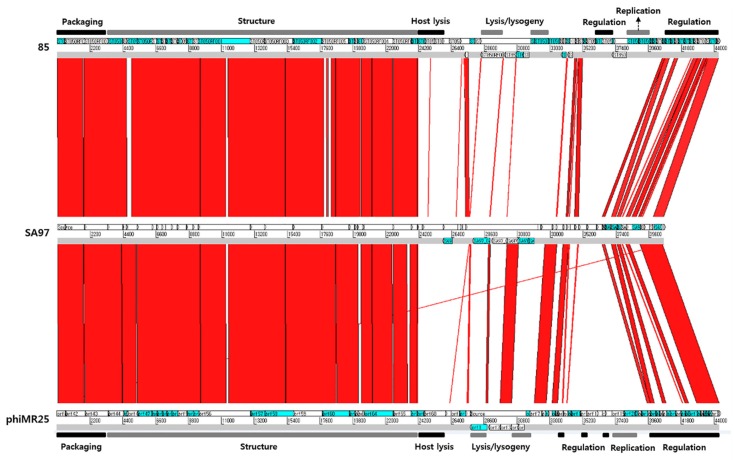
Comparative genomic analysis of phietalikevirus subgroup2 phages (SA97, 85 and phiMR25) with BLASTN and ACT12. Black and gray bars indicate the functions of gene clusters in the genomes.

A phylogenetic analysis of the terminase large subunits in the staphylococcal bacteriophages, including phage SA97, was performed (Figure 4). According to a previous study [46], Siphovirus staphylococcal phages cluster into three groups: “3alikevirus”, “77likevirus” and “phietalikevirus”. “phietalikevirus” includes 31 staphylococcal phages, which are theoretically divided again into three subgroups based on DNA similarity. The terminase large subunit of phage SA97 is similar to those of phage 85 [47] and phiMR25 [48], so that SA97 could be classified into the phietalikevirus subgroup2 as shown in Figure 4. Terminase large subunit of SA97 (SA97_011) has a terminase_3 domain like *B. subtilis* phage SPP1, which is known to use the *pac* site for DNA packaging [49], suggesting the possibility that SA97 would also use a *pac* mechanism for packaging DNA. Moreover, the tail morphogenesis module of SA97 consists of the SGNH hydrolase superfamily (SA97_026), tail protein (SA97_027), virion-associated peptidoglycan hydrolase (SA97_032) and tail fiber protein (SA97_033). The composition is the same as that of phages involved in “phietalikevirus subgroup2” as analyzed by Gutierrez *et al* [46]. Additional comparative genomic analysis of phage SA97 with phietalikevirus subgroup2 phages such as phage 85 and phage phiMR25 (Figure 5) revealed that DNA packaging and structure (heads and tails) gene clusters are highly conserved but the lysis/lysogeny control regions (Integrase, Repressor, Antirepressor and Cro-like protein) are not, suggesting that phage SA97 may have distinctive lytic/lysogenic decision mechanism even though phage SA97 is grouped in the phietalikevirus subgroup2 with phage 85 and phage phiMR25. The host cell lysis mechanism of SA97 is also expected to be different from those of phage 85 and phiMR25 because the endolysin and holin genes of SA97 differ from those of phage 85 and phiMR25.

**Table 2:** Functional categories of predicted open reading frames (ORFs) in bacteriophage SA97.

Group	Subgroup	Locus_tag ^a^	Function	BLASTP Best Match	Identity (%) ^b^	Accession no. ^c^
packaging		SA97_010	terminase small subunit	phage terminase small subunit [*Staphylococcus aureus* subsp. aureus JH1]	164/164 (100)	YP_001316047.1
packaging		SA97_011	terminase large subunit	PBSX family phage terminase large subunit [*Staphylococcus aureus* subsp. aureus JH9]	431/431 (100)	YP_001246264.1
packaging		SA97_012	portal protein	SPP1 family phage portal protein [*Staphylococcus aureus* subsp. aureus JH9]	512/512 (100)	YP_001246265.1
structure	head	SA97_013	head morphogenesis protein	phage head morphogenesis protein [*Staphylococcus aureus* subsp. aureus JH9]	331/331 (100)	YP_001246266.1
hypothetical		SA97_014	hypothetical protein	hypothetical protein phiETA2_gp45 [*Staphylococcus* phage phiETA2]	56/56 (100)	YP_001004305.1
structure	head	SA97_015	capsid protein	phage capsid protein [*Staphylococcus aureus*]	206/206 (100)	WP_000392146.1
structure	head	SA97_016	major capsid protein	Phage major capsid protein [*Staphylococcus aureus* subsp. aureus DSM 20231]	324/324 (100)	WP_001836233.1
hypothetical		SA97_017	hypothetical protein	phage protein [*Staphylococcus aureus* subsp. aureus DSM 20231]	95/95 (100)	ELP28728.1
structure	head-tail	SA97_018	head-tail adapter protein	head-tail connector protein [*Staphylococcus* phage SA12]	110/110 (100)	YP_008241892.1
hypothetical		SA97_019	hypothetical protein	hypothetical protein NWMN_1023 [*Staphylococcus aureus* subsp. aureus str. Newman]	115/115 (100)	YP_001332057.1
structure	head	SA97_020	head protein	hypothetical protein SAV0897 [*Staphylococcus aureus* subsp. aureus Mu50]	127/127 (100)	NP_371421.1
structure	tail	SA97_021	major tail protein	phage major tail protein [*Staphylococcus* phage phiNM]	193/193 (100)	YP_873998.1
hypothetical		SA97_022	hypothetical protein	hypothetical protein SaurJH9_0899 [*Staphylococcus aureus* subsp. aureus JH9]	121/121 (100)	YP_001246276.1
hypothetical		SA97_023	hypothetical protein	hypothetical protein T874_02808, partial [*Staphylococcus aureus* SJOS6126]	114/114 (100)	EVG26589.1
structure	tail	SA97_024	tape measure protein	putative tape measure protein [*Staphylococcus* phage phiSauS-IPLA88]	1130/1154 (98)	YP_002332525.1
structure	tail	SA97_025	putative minor tail protein	putative minor tail protein [*Staphylococcus* phage TEM123]	314/315 (99)	YP_ 006382275.1
structure	tail	SA97_026	prophage endopeptidase tail protein	SGNH hydrolase superfamily [*Staphylococcus aureus* subsp. aureus 21283]	632/632 (100)	EHO98287.1
structure	tail	SA97_027	putative minor tail protein	minor structural protein [*Staphylococcus aureus*]	635/636 (99)	WP_000369023.1
hypothetical		SA97_028	hypothetical protein	hypothetical protein NWMN_0306 [*Staphylococcus aureus* subsp. aureus str. Newman]	607/607 (100)	YP_001331340.1
hypothetical		SA97_029	hypothetical protein	SLT orf 129-like protein [*Staphylococcus aureus* subsp. aureus JH1]	125/125 (100)	YP_001316067.1
hypothetical		SA97_030	hypothetical protein	hypothetical protein SAP26_gp20 [*Staphylococcus* phage SAP-26]	57/57 (100)	YP_003857088.1
hypothetical		SA97_031	hypothetical protein	hypothetical protein phiETA2_gp63 [*Staphylococcus* phage phiETA2]	99/99 (100)	YP_001004323.1
structure	tail	SA97_032	mannosyl-glycoprotein endo-beta-*N*-acetylglucosminidase	mannosyl-glycoprotein endo-beta-*N*-acetylglucosaminidase [*Staphylococcus aureus* subsp. aureus DSM 20231]	632/632 (100)	WP_001836236.1
structure	tail	SA97_033	putative tail fiber protein	tail fiber [*Staphylococcus aureus* subsp. aureus JH1]	412/412 (100)	YP_001316071.1
hypothetical		SA97_034	hypothetical protein	hypothetical protein SASA_03740 [*Staphylococcus aureus* subsp. aureus DSM 20231]	131/131 (100)	ELP28745.1
host lysis		SA97_035	holin	holin, SPP1 family [*Staphylococcus aureus* subsp. aureus 21267]	91/91 (100)	EZH90125.1
host lysis		SA97_036	amidase	CHAP domain-containing protein [*Staphylococcus aureus* subsp. aureus JH9]	470/470 (100)	YP_001246290.1
hypothetical		SA97_037	hypothetical protein	hypothetical protein SaurJH1_0932 [*Staphylococcus aureus* subsp. aureus JH1]	185/185 (100)	YP_001316075.1
hypothetical		SA97_038	hypothetical protein	hypothetical protein SAOUHSC_02018 [*Staphylococcus aureus* subsp. aureus NCTC 8325]	50/50 (100)	YP_500515.1
regulation		SA97_039	putative transcriptional regulatory protein	XRE family transcriptional regulator [*Staphylococcus aureus*]	61/61 (100)	WP_000382163.1
lysis/lysogeny		SA97_040	putative integrase	integrase [*Staphylococcus aureus* subsp. aureus DSM 20231]	349/349 (100)	WP_001145725.1
hypothetical		SA97_041	hypothetical protein	hypothetical protein SAOUHSC_02087 [*Staphylococcus aureus* subsp. aureus NCTC 8325]	301/301 (100)	YP_500579.1
hypothetical		SA97_042	hypothetical protein	hypothetical protein phiETA2_gp04 [*Staphylococcus* phage phiETA2]	241/241 (100)	YP_001004264.1
hypothetical		SA97_043	hypothetical protein	hypothetical protein SaurJH1_0868 [*Staphylococcus aureus* subsp. aureus JH1]	224/224 (100)	YP_001316011.1
regulation		SA97_044	putative transcriptional regulator	XRE family transcriptional regulator [*Staphylococcus* *aureus*]	98/110 (89)	WP_001055143.1
lysis/lysogeny		SA97_045	anti-repressor	anti-repressor protein [*Staphylococcus* phage phiETA2]	255/255 (100)	YP_001004268.1
hypothetical		SA97_046	hypothetical protein	hypothetical protein WWS_01005 [*Staphylococcus aureus* M1510]	154/155 (99)	ENN21654.1|
hypothetical		SA97_047	hypothetical protein	hypothetical protein SAV0856 [*Staphylococcus aureus* subsp. aureus Mu50]	74/74 (100)	NP_371380.1
hypothetical		SA97_048	hypothetical protein	hypothetical protein phiETA2_gp10 [*Staphylococcus* phage phiETA2]	148/149 (99)	YP_001004270.1
hypothetical		SA97_049	hypothetical protein	hypothetical protein phiETA3_gp11 [*Staphylococcus* phage phiETA3]	73/73 (100)	YP_001004340.1
hypothetical		SA97_050	hypothetical protein	hypothetical protein SaurJH1_0876 [*Staphylococcus aureus* subsp. aureus JH1]	100/100 (100)	YP_001316019.1
hypothetical		SA97_051	hypothetical protein	hypothetical protein SASA_03250 [*Staphylococcus aureus* subsp. aureus DSM 20231]	86/86 (100)	ELP28696.1
hypothetical		SA97_052	hypothetical protein	hypothetical protein phiSLTp16 [*Staphylococcus* phage phiSLT]	177/178 (99)	NP_075479.1
regulation		SA97_053	single-stranded DNA-binding protein 1	ssDNA-binding protein *St aphylococcus aureus* SA_ST125_MupR]	216/216 (100)	EPR23947.1
regulation		SA97_054	single-stranded DNA-binding protein 2	ssDNA-binding protein [*Staphylococcus* phage phiETA3]	104/105 (99)	YP_001004346.1
hypothetical		SA97_001	hypothetical protein	hypothetical protein U622_02779, partial [*Staphylococcus aureus* F53399]	61/61 (100)	EWV07862.1
hypothetical		SA97_002	hypothetical protein	hypothetical protein SaurJH9_0873 [*Staphylococcus aureus* subsp. aureus JH9]	101/101 (100)	YP_001246250.1
hypothetical		SA97_003	hypothetical protein	hypothetical protein phiETA3_gp28 [*Staphylococcus* phage phiETA3]	118/118 (100)	YP_001004357.1
hypothetical		SA97_004	hypothetical protein	hypothetical protein SASA_03390 [*Staphylococcus aureus* subsp. aureus DSM 20231]	80/80 (100)	ELP28710.1
hypothetical		SA97_005	hypothetical protein	phage protein [*Staphylococcus aureus* M0157]	110/110 (100)	EUU37240.1
regulation		SA97_006	dUTP diphosphatase	dUTP diphosphatase [*Staphylococcus aureus* A9765]	175/176 (99)	WP_001797468.1
hypothetical		SA97_007	hypothetical protein	hypothetical protein [*Staphylococcus* phage phiMR25]	67/68 (99)	YP_001949833.1
regulation		SA97_008	transcriptional activator RinB	transcriptional activator RinB [*Staphylococcus aureus* subsp. aureus CM05]	57/57 (100)	WP_001657250.1
regulation		SA97_009	transcriptional activator RinA	transcriptional activator RinA [*Staphylococcus* phage phiETA3]	140/140 (100)	YP_001004368.1

^a^, Each predicted ORFs of SA97 phage was labeled with locus_tag. ^b^, Identities were calculated between amino acid sequences. ^c^, Genbank accession numbers.

### 3.6. Determination of the Frequency of BIMs and Horizontal Gene Transfer by SA97

The frequencies of BIMs were calculated with SA97 and SA12 phages. *S. aureus* temperate phage SA12 [17] was compared because the temperate phage is generally known to show a higher frequency of BIMs compared to the lytic phages [27]. The frequency of BIMs of *S. aureus* RN4220 by SA97 was 4.4 × 10^−6^ ± 2.9 × 10^−6^, which was much lower than 2.8 × 10^−3^ ± 2.79 × 10^−3^ observed by SA12 phage. These results indicate that the possibility of lysogen formation by SA97 is very low compared with SA12.

To evaluate the ability of the SA97 to transduce, frequencies of erythromycin-resistant gene transduction by SA97 were calculated with *S. aureus* RN4220 and four isolates (Table 3). SA12 phage was used as a positive control. No erythromycin-resistant colonies were found from any strain tested after transduction with SA97, suggesting that the horizontal gene transfer by SA97 may have hardly occurred. However, the transduction was possible with SA12 with transduction frequencies ranging from 1.7 × 10^−6^ ± 1.2 × 10^−8^ to 6.0 × 10^−7^ ± 1.6 × 10^−7^. We do not understand the cause of this difference because most *pac*-type phages such as SA12 and SA97 are known to be able to transduce bacterial DNA, but it is speculated that SA12 may transduce better than SA97 because SA12 can produce lysogen while SA97 cannot. Genome analysis revealed that SA12 has intact lysogeny-related genes [recombinase (SA12_ORF07), *cI*-like repressor (SA12_ORF54), *cro*-like repressor (SA12_ORF55) and antirepressor (SA12_ORF57)] but that *cI*-like repressor, a key gene of the lysogeny module, is missing in the phage SA97 genome.

**Table 3:** Transduction frequencies with phages SA97 and SA12.

Recipient Strain	Transduction	Frequency
	SA97	SA12
*S. aureus* RN4220	0.0 ± 0.0	1.7 × 10^−8^± 1.2 × 10^−8^
*S. aureus* food isolate 1	0.0 ± 0.0	2.6 × 10^−7^± 1.5 × 10^−7^
*S. aureus* food isolate 2	0.0 ± 0.0	6.0 × 10^−7^± 1.6 × 10^−7^
*S. aureus* food isolate 3	0.0 ± 0.0	5.1 × 10^−8^± 1.2 × 10^−8^
*S. aureus* clinical isolate 1	0.0 ± 0.0	1.3 × 10^−7^± 0.5 × 10^−7^

In conclusion, SA97 could not cause horizontal gene transfer despite containing genes encoding a lysogeny module, likely because the phage lacks a key gene (*cI*) important for lysogen formation. Our results suggest that even phages containing a lysogen module should be examined for their ability to transduce or lysogenize because they can be used as biocontrol agents if they can form neither lysogen nor transductant.

## References

[1] Mahony J., McAuliffe O., Ross R.P., van Sinderen D. (2011). Bacteriophages as biocontrol agents of food pathogens. Curr. Opin. Biotech..

[2] Lowy F.D. (1998). *Staphylococcus aureus* infections. N. Engl. J. Med..

[3] Bal A.M., Gould I.M. (2005). Antibiotic resistance in *Staphylococcus aureus* and its relevance in therapy. Expert Opin. Pharmacother..

[4] Appelbaum P.C. (2006). The emergence of vancomycin-intermediate and vancomycin-resistant *Staphylococcus aureus*. Clin. Microbiol. Infect..

[5] Sulakvelidze A., Alavidze Z., Morris J.G. (2001). Bacteriophage therapy. Antimicrob. Agents Chemother..

[6] Lang L.H. (2006). FDA approves use of bacteriophages to be added to meat and poultry products. Gastroenterology.

[7] Guenther S., Huwyler D., Richard S., Loessner M.J. (2009). Virulent bacteriophage for efficient biocontrol of *Listeria monocytogenes* in ready-to-eat foods. Appl. Environ. Microb..

[8] Matsuzaki S., Yasuda M., Nishikawa H., Kuroda M., Ujihara T., Shuin T., Shen Y., Jin Z., Fujimoto S., Nasimuzzaman M.D. (2003). Experimental protection of mice against lethal *Staphylococcus aureus* infection by novel bacteriophage phiMR11. J. Infect. Dis..

[9] O’Flaherty S., Ross R.P., Meaney W., Fitzgerald G.F., Elbreki M.F., Coffey A. (2005). Potential of the polyvalent anti-*Staphylococcus* bacteriophage K for control of antibiotic-resistant staphylococci from hospitals. Appl. Environ. Microbiol..

[10] O'Flaherty S., Ross R.P., Flynn J., Meaney W.J., Fitzgerald G.F., Coffey A. (2005). Isolation and characterization of two anti-staphylococcal bacteriophages specific for pathogenic *Staphylococcus aureus* associated with bovine infections. Lett. Appl. Microbiol..

[11] Takemura-Uchiyama I., Uchiyama J., Kato S., Inoue T., Ujihara T., Ohara N., Daibata M., Matsuzaki S. (2013). Evaluating efficacy of bacteriophage therapy against *Staphylococcus aureus* infections using a silkworm larval infection model. FEMS Microbiol. Lett..

[12] Goerke C., Pantucek R., Holtfreter S., Schulte B., Zink M., Grumann D., Broker B.M., Doskar J., Wolz C. (2009). Diversity of prophages in dominant *Staphylococcus aureus* clonal lineages. J. Bacteriology.

[13] Blair J.E., Carr M. (1961). Lysogeny in staphylococci. J. Bacteriol..

[14] Simatake H., Rosenberg M. (1981). Purified lambda regulatory protein CII positively activates promoters for lysogenic development. Nature.

[15] Ptashne M., Hopkins N. (1968). The operators controlled by the lambda phage repressor. Proc. Natl. Acad. Sci. USA.

[16] Groth A.C., Calos M.P. (2004). Phage integrases: Biology and applications. J. Mol. Biol..

[17] Chang Y., Lee J.H., Shin H., Heu S., Ryu S. (2013). Characterization and complete genome sequence analysis of *Staphylococcus aureus* bacteriophage SA12. Virus Genes.

[18] Kwiatek M., Parasion S., Mizak L., Gryko R., Bartoszcze M., Kocik J. (2012). Characterization of a bacteriophage, isolated from a cow with mastitis, that is lytic against *Staphylococcus aureus* strains. Arch. Virol..

[19] Rodhain F., Francki R.I.B., Fauquet C.M., Knudson D.I., Brown F. (1995). Classification and Nomenclature of Viruses—5th Report of the International Committee for Virus Taxonomy.

[20] Park M., Lee J.H., Shin H., Kim M., Choi J., Kang D.H., Heu S., Ryu S. (2012). Characterization and comparative genomic analysis of a novel bacteriophage, SFP10, simultaneously inhibiting both *Salmonella enterica* and *Escherichia coli* O157:H7. Appl. Environ. Microb..

[21] Lu Z., Breidt F., Fleming H.P., Altermann E., Klaenhammer T.R. (2003). Isolation and characterization of a *Lactobacillus plantarum* bacteriophage, phiJL-1, from a cucumber fermentation. Int. J. Food Microbiol..

[22] Kreiswirth B.N., Lofdahl S., Betley M.J., O’Reilly M., Schlievert P.M., Bergdoll M.S., Novick R.P. (1983). The toxic shock syndrome exotoxin structural gene is not detectably transmitted by a prophage. Nature.

[23] Wann E.R., Dassy B., Fournier J.M., Foster T.J. (1999). Genetic analysis of the cap5 locus of *Staphylococcus aureus*. FEMS Microbiol. Lett..

[24] Swoboda J.G., Campbell J., Meredith T.C., Walker S. (2010). Wall teichoic acid function, biosynthesis, and inhibition. Chembiochem.

[25] Bruckner R. (1992). A series of shuttle vectors for *Bacillus subtilis* and *Escherichia coli*. Gene.

[26] Yoon H., Yun J., Lim J.A., Roh E., Jung K.S., Chang Y., Ryu S., Heu S. (2013). Characterization and genomic analysis of two *Staphylococcus aureus* bacteriophages isolated from poultry/livestock farms. J. Gen. Virol..

[27] Garcia P., Madera C., Martinez B., Rodriguez A. (2007). Biocontrol of *Staphylococcus aureus* in curd manufacturing processes using bacteriophages. Int. Dairy J..

[28] Kiriukhin M.Y., Debabov D.V., Shinabarger D.L., Neuhaus F.C. (2001). Biosynthesis of the glycolipid anchor in lipoteichoic acid of *Staphylococcus aureus* RN4220: Role of YpfP, the diglucosyldiacylglycerol synthase. J. Bacteriol..

[29] Welker N.E. (1988). Transduction in *Bacillus stearothermophilus*. J. Bacteriol..

[30] Wilcox S.A., Toder R., Foster J.W. (1996). Rapid isolation of recombinant lambda phage DNA for use in fluorescence *in situ* hybridization. Chromosome Res..

[31] Delcher A.L., Bratke K.A., Powers E.C., Salzberg S.L. (2007). Identifying bacterial genes and endosymbiont DNA with Glimmer. Bioinformatics.

[32] Lukashin A.V., Borodovsky M. (1998). GeneMark.hmm: New solutions for gene finding. Nucleic Acids Res..

[33] Ziedaite G., Daugelavicius R., Bamford J.K., Bamford D.H. (2005). The Holin protein of bacteriophage PRD1 forms a pore for small-molecule and endolysin translocation. J. Bacteriol..

[34] Altschul S.F., Madden T.L., Schaffer A.A., Zhang J., Zhang Z., Miller W., Lipman D.J. (1997). Gapped BLAST and PSI-BLAST: A new generation of protein database search programs. Nucleic Acids Res..

[35] Altschul S.F., Gish W., Miller W., Myers E.W., Lipman D.J. (1990). Basic local alignment search tool. J. Mol. Biol..

[36] Carver T., Berriman M., Tivey A., Patel C., Bohme U., Barrell B.G., Parkhill J., Rahandream M.A. (2008). Artemis and ACT: Viewing, annotating and comparing sequences stored in a relational database. Bioinformatics.

[37] Deghorain M., Bobay L.M., Smeesters P.R., Bousbata S., Vermeersch M., Perez-Morga D., Dreze P.A., Rocha E.P., Touchon M., van Melderen L. (2012). Characterization of novel phages isolated in coagulase-negative staphylococci reveals evolutionary relationships with *Staphylococcus aureus* phages. J. Bacteriol..

[38] Guoqing X., Christiane W. (2014). Phages of *Staphylococcus aureus* and their impact on host evolution. Infect. Genet. Evol..

[39] Han J.E., Kim J.H., Hwang S.Y., Choresca C.H., Shin S.P., Jun J.W., Chai J.Y., Park Y.H., Park S.C. (2013). Isolation and characterization of a *Myoviridae* bacteriophage against *Staphylococcus aureus* isolated from dairy cows with mastitis. Res. Vet. Sci..

[40] Xia G., Corrigan R.M., Winstel V., Goerke C., Grundling A., Peschel A. (2011). Wall teichoic Acid-dependent adsorption of staphylococcal siphovirus and myovirus. J. Bacteriol..

[41] Oku Y., Kurokawa K., Matsuo M., Yamada S., Lee B.L., Sekimizu K. (2009). Pleiotropic roles of polyglycerolphosphate synthase of lipoteichoic acid in growth of *Staphylococcus aureus* cells. J. Bacteriol..

[42] Park K.H., Kurokawa K., Zheng L., Jung D.J., Tateishi K., Jin J.O., Ha N.C., Kang H.J., Matsushita M., Kwak J.Y. (2010). Human serum mannose-binding lectin senses wall teichoic acid Glycopolymer of *Staphylococcus aureus*, which is restricted in infancy. J. Biol. Chem..

[43] Baba T., Bae T., Schneewind O., Takeuchi F., Hiramatsu K. (2008). Genome sequence of *Staphylococcus aureus* strain Newman and comparative analysis of staphylococcal genomes: Polymorphism and evolution of two major pathogenicity islands. J. Bacteriol..

[44] Loessner M.J. (2005). Bacteriophage endolysins-current state of research and applications. Curr. Opin. Microbiol..

[45] Rodriguez-Rubio L., Martinez B., Donovan D.M., Rodriguez A., Garcia P. (2013). Bacteriophage virion-associated peptidoglycan hydrolases: Potential new enzybiotics. Crc. Cr. Rev. Microbiol..

[46] Gutierrez D., Adriaenssens E.M., Martinez B., Rodriguez A., Lavigne R., Kropinski A.M., Garcia P. (2014). Three proposed new bacteriophage genera of staphylococcal phages: “3alikevirus”, “77likevirus”, “Phietalikevirus”. Arch. Virol..

[47] Kwan T., Liu J., DuBow M., Gros P., Pelletier J. (2005). The complete genomes and proteomes of 27 *Staphylococcus aureus* bacteriophages. Proc. Natl. Acad. Sci. USA.

[48] Hoshiba H., Uchiyama J., Kato S., Ujihara T., Muraoka A., Daibata M., Wakiguchi H., Matsuzaki S. (2010). Isolation and characterization of a novel *Staphylococcus aureus* bacteriophage, phiMR25, and its therapeutic potential. Arch. Virol..

[49] Chai S., Bravo A., Luder G., Nedlin A., Trautner T.A., Alonso J.C. (1992). Molecular analysis of the *Bacillus subtilis* bacteriophage SPP1 region encompassing genes 1 to 6. J. Mol. Biol..

